# Supplementary material for: A slit-diaphragm-associated protein network for dynamic control of renal filtration
Source: Nat Commun. 2022 Oct 28;13:6446. doi: 10.1038/s41467-022-33748-1 (PMC9616960; doi:10.1038/s41467-022-33748-1)
Supplement: Supplementary file 1 — Supplementary Information [file 41467_2022_33748_MOESM1_ESM.pdf]

Supplemental Material

A slit-diaphragm-associated protein network for dynamic control of renal filtration

Maciej K. Kocylowski, Hande Aypek, Wolfgang Bildl, Martin Helmstädter, Philipp Trachte, Bernhard Dumoulin, Sina Wittösch, Lukas Kühne, Ute Aukschun, Oliver Kretz, Botond Gaal, Akos Kulik, Corinne Antignac, Geraldine Mollet, Anna Köttgen, Burulca Göcmen, Jochen Schwenk, Uwe Schulte, Tobias B. Huber, Bernd Fakler and Florian Grahammer

- Supplementary Figures 1 – 9

Supplementary Figure 1 (information related to Figure 1)

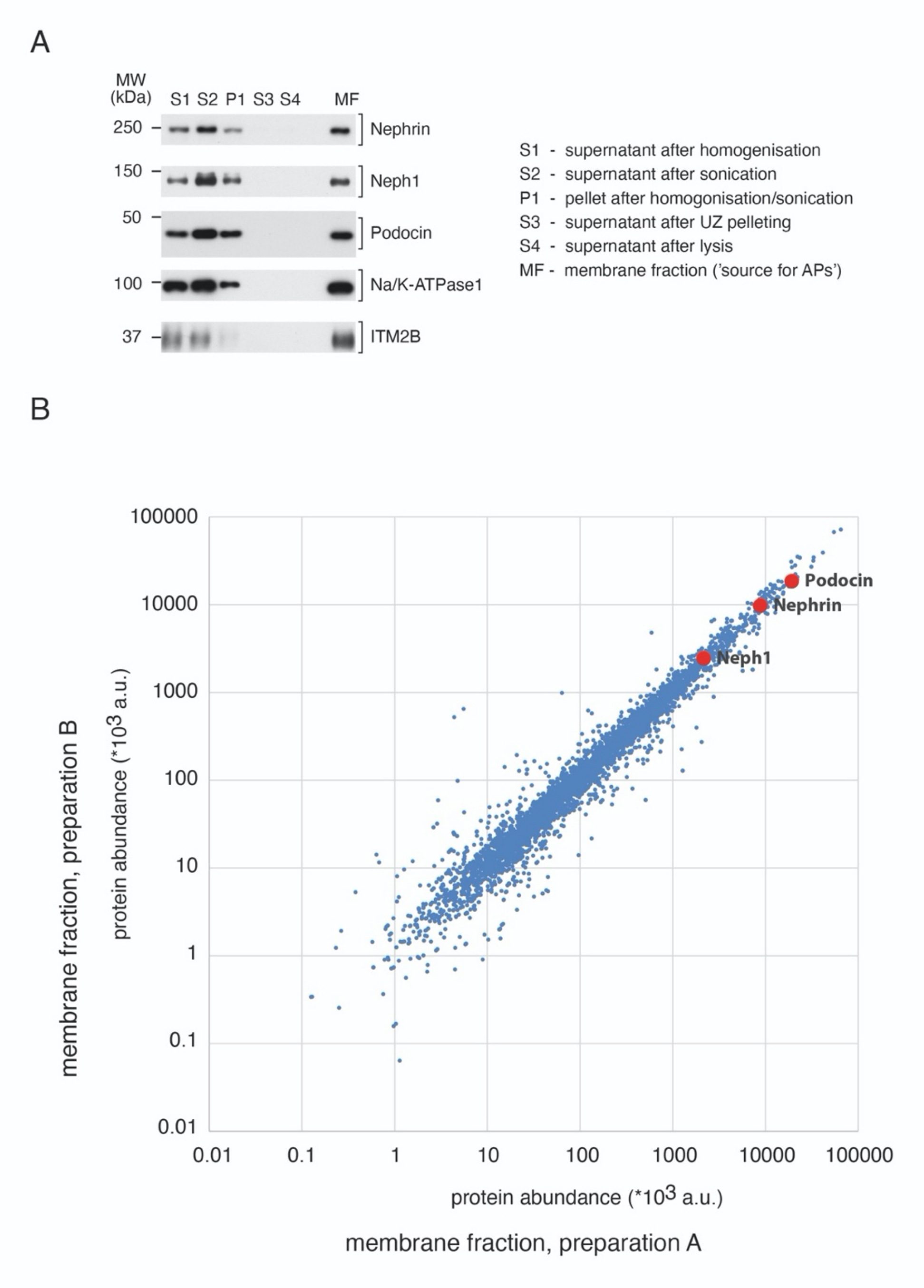

Supplementary Figure 1
Characterization of glomerular membrane fractions
(**A**), Denaturing gel separation of the protein fractions obtained during preparation of the glomerular 'membrane fraction' (MF) used for meAPs Western-probed for the indicated proteins. The alpha1-subunit of the Na-K-ATPase was used a marker for plasma membrane proteins. Experiment was repeated at least four times. (**B**), Abundance of all proteins determined by MS-analysis in two independent preparations of the MF-fraction. Note enrichment of the SD core components in either preparation.

Supplementary Figure 2 (information related to Figures 1, 2)

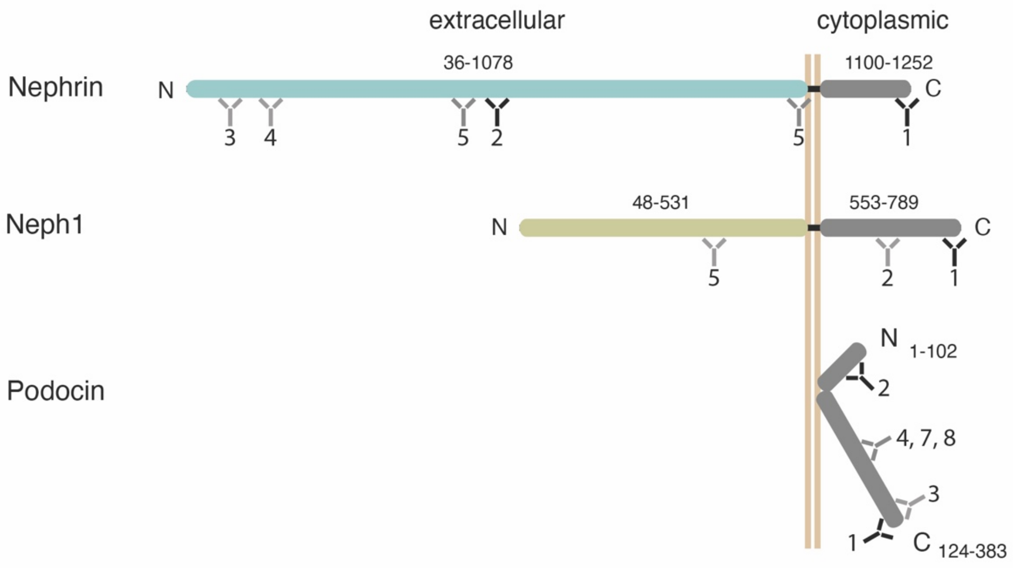

target	no.	supplier	cat. No.	epitope/antigen	source	efficiency
**Nephrin**	**a1**	Progen	GP-N2	1243-1256	Progen	1.00
**Nephrin**	**a2**	R&D Systems	AF3159	37-1049	R&D Systems	0.70
Nephrin	a3	Santa-Cruz	sc19000	N-terminus	Santa-Cruz	0.07
Nephrin	a4	Santa-Cruz	sc28192	23-322	Santa-Cruz	0.00
Nephrin	a5	AbFrontier	pep3+6	491-509/1053-1073	this study	WB -
**Neph1**	**a1**	Pineda		767-788	Grahammer et al.	1.00
Neph1	a2	AbFrontier	pep3	658-678	this study	0.48
Neph1	a3	AbFrontier	R2	365-387/658-678	this study	0.12
Neph1	a4	AbFrontier	R1	365-387/658-678	this study	0.10
Neph1	a5	AbFrontier	pep1	365-387	this study	0.01
**Podocin**	**a1**	sigma	P0372	367-383	sigma	1.00
**Podocin**	**a2**	AbFrontier	pep1	34-47	this study	0.54
Podocin	a3	AbFrontier	pep3	354-368	this study	0.17
Podocin	a4	AbFrontier	C-term R1	135-383	Roselli et al.	0.07
Podocin	a5	AbFrontier	R1	34-47/354-368	this study	0.05
Podocin	a6	AbFrontier	R2	34-47/354-368	this study	0.01
Podocin	a7	AbFrontier	C-term R2	135-383	Roselli et al.	WB+
Podocin	a8	AbFrontier	C-term R3	135-383	Roselli et al.	WB+

Supplementary Figure 2
Characterization of antibodies
Epitope-localization and characterization of ABs against the SD core components Nephrin, Neph1 and Podocin. The ABs highlighted in bold for each target were finally used for the meAPs reported in this work. Efficiency reflects the relative abundance of the respective target protein in APs determined by MS-analysis and normalized to the best performing AB. WB -/+ indicates that antibody efficiency was tested only by AP-WB.

Supplementary Figure 3 (information related to Figures 1, 2)

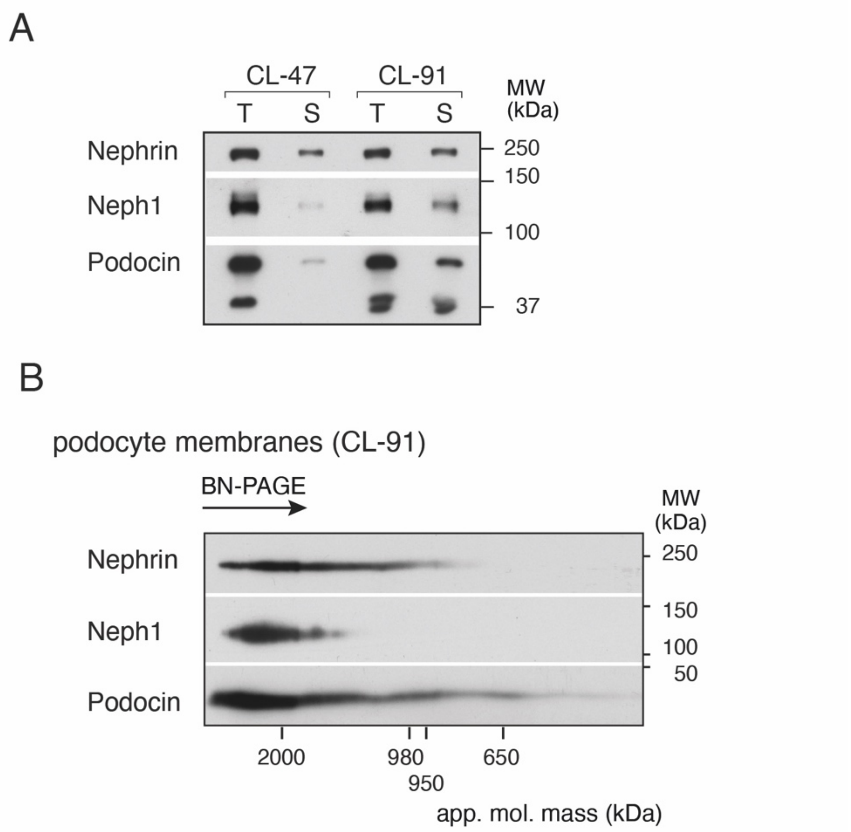

Supplementary Figure 3
Solubilization efficiency and gel separation of CL-91 solubilized membrane fractions
(**A**), SDS-PAGE separation of unsolubilized (T) and CL-solubilized (S) membrane fractions Western-probed for the indicated SD core constituents; MW scaling as indicated. Experiment was at least five times. (**B**), Two-dimensional gel separation of CL-91 solubilized membrane fractions prepared from isolated rat glomeruli, Western-probed with ABs against the indicated proteins. Apparent molecular mass (native PAGE, 1st dimension) and molecular weight (denaturing SDS-PAGE, 2nd dimension) are indicated; gel separations were repeated twice with similar results.

Supplementary Figure 4 (next page, information related to Figures 1, 2)

Coverage of primary sequences and phosphorylation status of the indicated SD core components derived from MS/MS-identified peptides
Left panel: Peptides identified by mass spectrometry are in red; those accessible to but not identified in MS/MS analyses are in black, and peptides hardly or not accessible to our MS/MS analyses used are given in grey. Phosphorylated residues are highlighted in yellow; transmembrane segment is indicated by a line.
Right panel: Exemplary MS/MS spectra (b- and y-ion series) of the indicated phosphorylated peptides of Nephrin (top), Neph1 (middle) and Podocin (bottom).

Note the ‘triple phosphorylation’ occurring in the ‘acidic cluster’ C-terminal to the transmembrane domain of Nephrin.

NPHN_RAT (Q9R044) - Nephrin
Coverage is **45.4%** absolute, **64.2%** relative.

```
0001 MGAKRVTVRG ARTSPIHRMS SLTPLLLMGM LTSGLAESPV PTSAPRGFWA
0051 LSENLTAVEG TTVKLWCGVR APGSVVQWAK DGLLLGPNPK MPGFPRYSLE
0101 GDRAKGEFHL LIEACDLSDD AEYECQVGRS ELGPELVSPK VILSILVSPK
0151 VLLLTPEAGS TVTWVAGQEY VVTCVSGDAK PAPDITFIQS GRTILDVSSN
0201 VNEGSEEKLC ITEAEARVIP QSSDNGQLLV CEGSNPALDT PIKASFTMNI
0251 LFPPGPPVID WPGLNEGHVR AGENLELPCT ARGGNPPATL QWLKNGKPVS
0301 TAWGTEHAQA VAHSVLVMTV RPEDHGARLS CQSYNSVSAG TQERSITLQV
0351 TFPPSAITIL GSVSQSENKN VTLCCLTKSS RPRVLLRWWL GGRQLLPTDE
0401 TVMDGLHGGH ISMSNLTFLV RREDNGLPLT CEAFSDAFSK ETFKKSLTLN
0451 VKYPAQKLWI EGPPEGQYIR TGTRVRLVCL AIGGNPDPSL IWFKDSRPVS
0501 EPRQPQEPRR VQLGSVEKSG STFSRELVLI IGPPDNRAKF SCKAGQLSAS
0551 TQLVVQFPPT NLTILANSSA LRPGDALNLT CVSISSNPPV NLSWDKEGER
0601 LEDVAAKPQS APFKGSAASR SVFLRVSSRD HGQRVTCRAH SEALRETVSS
0651 FYRFNVLYPP EFLGEQVRAV TVVEQGQVLL PVSVSANPAP EAFNWTFRGY
0701 RLSPAGGPRH RILSGGALQL WNVTRADDGF YQLHCQNSEG TAEALLKLDV
0751 HYAPTIRALR DPTEVNVGGS VDIVCTVDAN PILPEMFSWE RLGEEEEDLN
0801 LDDMEKVSKG STGRLRIRQA KLSQAGAYQC IVDNGVAPAA RGLVRLVVRF
0851 APQVDQPTPL TKVAAAGDST SSATLHCRAR GVPNIDFTWT KNGVPLDLQD
0901 PRYTEHRYHQ GVVHSSLLTI ANVSAAQDYA LFKCTATNAL GSDHTNIQLV
0951 SISRPDPPLG LKVVSISPHS VGLEWKPGFD GGLPQRFQIR YEALETPGFL
1001 HVDVLPTQAT TFTLTGLKPS TRYRIWLLAS NALGDSGLTD KGIQVSVTTP
1051 GPDQAPEDTD HQLPTELPPG PPRLPLLPVL FAVGGLLLLS NASCVGGLLW
1101 RRRLRRLAEE ISEKTEAGSE DRIRNEYEES QWTGDRDTRS STVSTAEVDP
1151 NYYSMRDFSP QLPPTLEEVL YHQGAEGEDM AFPGHLHDEV ERAYGPPGAW
1201 GPLYDEVRMD PYDLRWPEVQ CEDPRGIYDQ VAADMDAVEA SSLPFELRGH
1251 LV
```

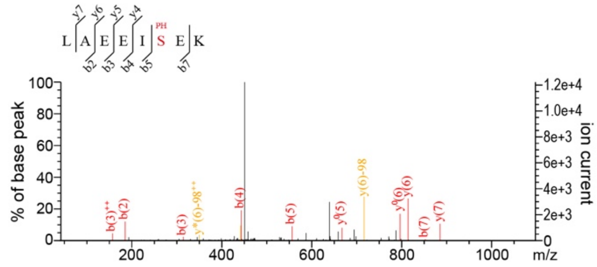

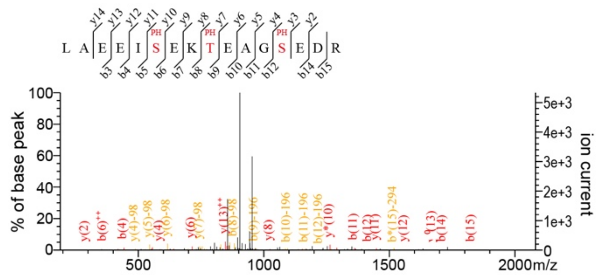

KIRR1_RAT (Q6X936) - Kin Of IRRE-like Protein 1
Coverage is **49.9%** absolute, **68.9%** relative.

```
0001 MTLENRSTCL MTCQSSLLPK KPRFLSQKMW APHLVVAYLI FVTLALALPG
0051 TQTRFSQEPA DQTVVAGHRA VLPCVLLNYS GIVQWTKDGL ALGMGQGLKA
0101 WPRYRVVGSA DAGQYNLEIT DAELSDDASY ECQATEAALR SRRAKLTVLI
0151 PPEDTRIDGG PVILLQAGTP YNLTCRAFNA KPAATIIWFR DGTQQEGAVT
0201 STELLKDGKR ETTISQLLIQ PTDLDIGRVF TCRSMNEAIP NGKETSIELD
0251 VHHPPTVTLS IEPQTVLEGE RVIFTCQATA NPEILGYRWA KGGFLIEDAH
0301 ESRYETNVDY SFFTEPVSCE VYNKVGSTNV STLVNVHFAP RIVVYPKPTT
0351 TDIGSDVTLT CVWVGNPPLT LTWTKKDSNM VLSNSNQLLL KSVTQADAGT
0401 YTCRAIVPRI GVAEREVPLY VNGPPIISSE AVQFAVRGDG GKVECFIGST
0451 PPPDRIAWAW KENFLEVGTL ERYTVERTNS GSGVLSTLTI NNVMEADFQT
0501 HYNCTAWNSF GPGTAIIQLE EREVLPVGII AGATIGAGIL LVFSFAALVF
0551 FLYRRRKGSR KDVTLRKLDI KVETVNREPL TMHSDREDDT TSISTATRVM
0601 KAIYSSFKDD VDLKQDLHCD TIETREEYEM KDPTNGYYNV RAHEDRPSSR
0651 AVLYADYRAP GPTRFDGRPS SRLSHSSGYA QLNTYSRAPA SDYGTEPTPS
0701 GPSAPGGTDT TSQLSYENYE KFNSHPFPGA AGYPTYRLGY PQAPPSGLER
0751 TPYEAYDPIG KYATATRFSY TSQHSDYGQR FQQRMQTHV
```

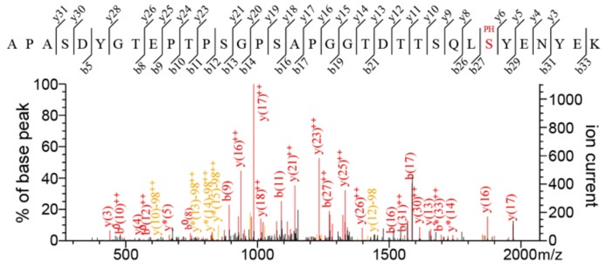

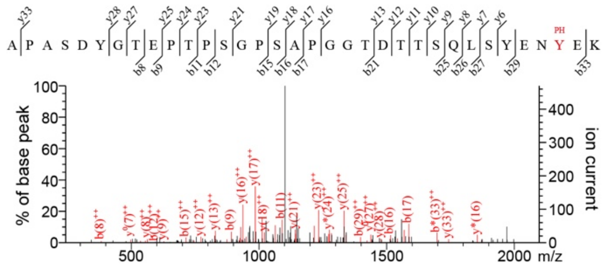

	undetectable	indirectly findable	findable
found		SEQUENCE	SEQUENCE
not found	SEQUENCE	SEQUENCE	SEQUENCE

PODO_MOUSE (Q91X05) - Podocin
Coverage is **65.5%** absolute, **91.6%** relative.

```
0001 MDSRARSSSR EAHGRSSRSS SRDDKKAKAG RGSRGRARPD AGAERQSTGR
0051 TATRGEPRAP AATATVVDVD EVRGPGEEGT EVVALLESER PEEGIKPSGL
0101 GACEWLLVLA SLIFIIMTFP FSIWFCIKVV QEYERVIIFR LGHLLPGRAK
0151 GPGLFFFLPC LDTYHKVDLR LQTLEIPFHE VVTKDMFIME IDAVCYYRME
0201 NASLLLSSLA HVSKAIQFLV QTTMKRLLAH RSLTEILLER KSIAQDVKVA
0251 LDAVTCIWGI KVERTEIKDV RLPAGLQHSL AVEAEAQRQA KVRVIAAEGE
0301 KAASESLRMA AEILSGTPAA VQLRYLHTLQ SLSTEKPATV VLPLPFDMLS
0351 LLSSPGNRAQ GSINYPSSSK PVEPLNPKKK DSPML
```

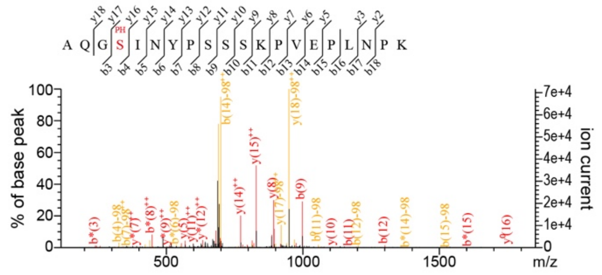

Supplementary Figure 4

Supplementary Figure 5 (information related to Figures 1, 2)

target-normalized ratio (tnR)

$$tnR = \frac{Log_{10}\left(ratio_{protein} / ratio_{mean,\ background}\right)}{Log_{10}\left(ratio_{target} / ratio_{mean,\ background}\right)}$$

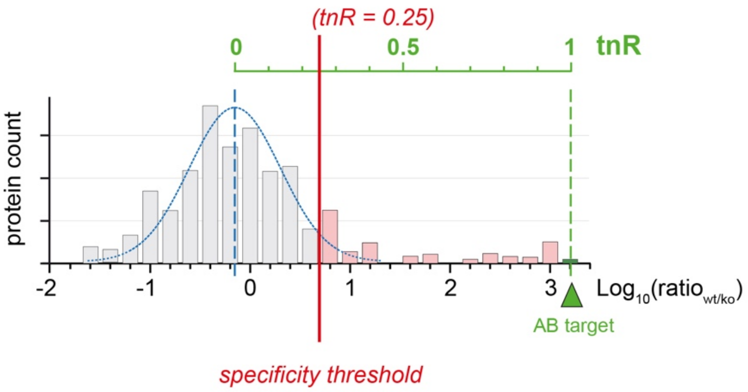

Supplementary Figure 5
Target-normalization of protein abundance ratios in APs versus control
Upper panel: Formula used for calculating tnR from respective protein ratio, target protein ratio and the mean of background protein ratios in log space. Lower panel: Histogram illustrating an example distribution of protein ratios in AP vs control (arbitrary values) together with the determination of the specificity threshold (tnR = 0.25). Distribution of ratios determined for background proteins is indicated by a blue line (fit of a Gaussian distribution, peak close to 0 in log space), the ratio of the AP target protein is given by the triangle in green. Note that normalization of protein ratios is prerequisite for direct comparison of results from APs with different antibodies and control IgGs.

Supplementary Figure 6 (next page, information related to Figure 3)

Characterization of glomeruli and podocytes in *Nphs1*$^{\Delta iPod}$, *Nphs2*$^{\Delta iPod}$ and *Neph1*$^{\Delta iPod}$ knockout mice
(**A**), Immuno-staining of podocytes (from three animals each) for Nephrin, Neph1 and Podocin (left panels), PAS-staining of kidney tissue and EM analysis of FPs in wildtype (control), *Nphs1*$^{\Delta iPod}$ and *Neph1*$^{\Delta iPod}$ knockout mice 4 weeks after induction by doxycycline. *Nphs1*$^{\Delta iPod}$ showed no changes in immuno-staining pattern nor in glomerulus morphology compared to wildtype. In *Neph1*$^{\Delta iPod}$ mice Neph1-staining was slightly reduced at unchanged PAS staining, podocyte FPs appeared widely flattened in line with the pronounced albuminuria (Fig. 3B). (**B**), In *Nphs2*$^{\Delta iPod}$ mice immuno-staining (from three animals each) was reduced for all three SD core proteins, PAS stain indicated segmental loss of the glomerular tuft with extensive synechia of Bowman's capsule. In addition, the FPs were largely flattened correlating with the massive albuminuria (Fig. 3C).

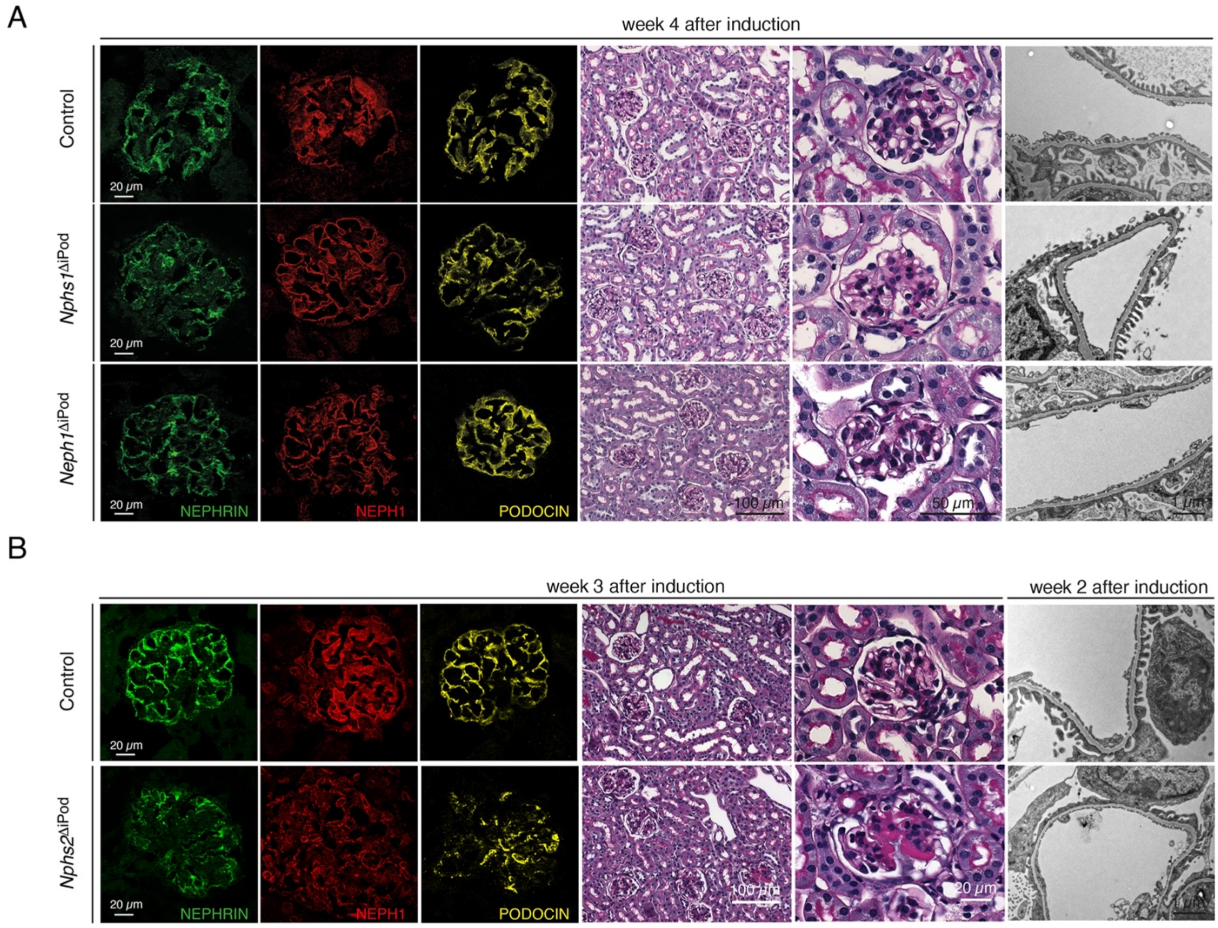

Supplementary Figure 6

Supplementary Figure 7 (next page, information related to Figure 3)

Alterations in the SD-associated network following induced knock-down of the SD core-components

Protein abundances were determined in (unsolubilized) total membrane fractions by mass spectrometry.

(**A**), Abundance ratios (log10) determined for the indicated constituents of the Nephrin interactome (relative to controls (without doxycycline administration, 'WT') at distinct periods (1 week (green), 6 weeks (purple) and 12 weeks (orange)) after induction of Nephrin KO (Nphs1$^{\Delta iPod}$). (**B**), Abundance ratios as in the top panel determined 4 weeks after induction of Neph1 KO (Neph1$^{\Delta iPod}$). (**C**), Abundance changes determined for the indicated constituents of the Podocin interactome 2 weeks after induction of Podocin KO (Nphs2$^{\Delta iPod}$). (**D**), Overlay of the changes determined for the common SD-core interactors at the indicated time points after KO induction. Note consistency and interdependence in stability of these SD constituents.

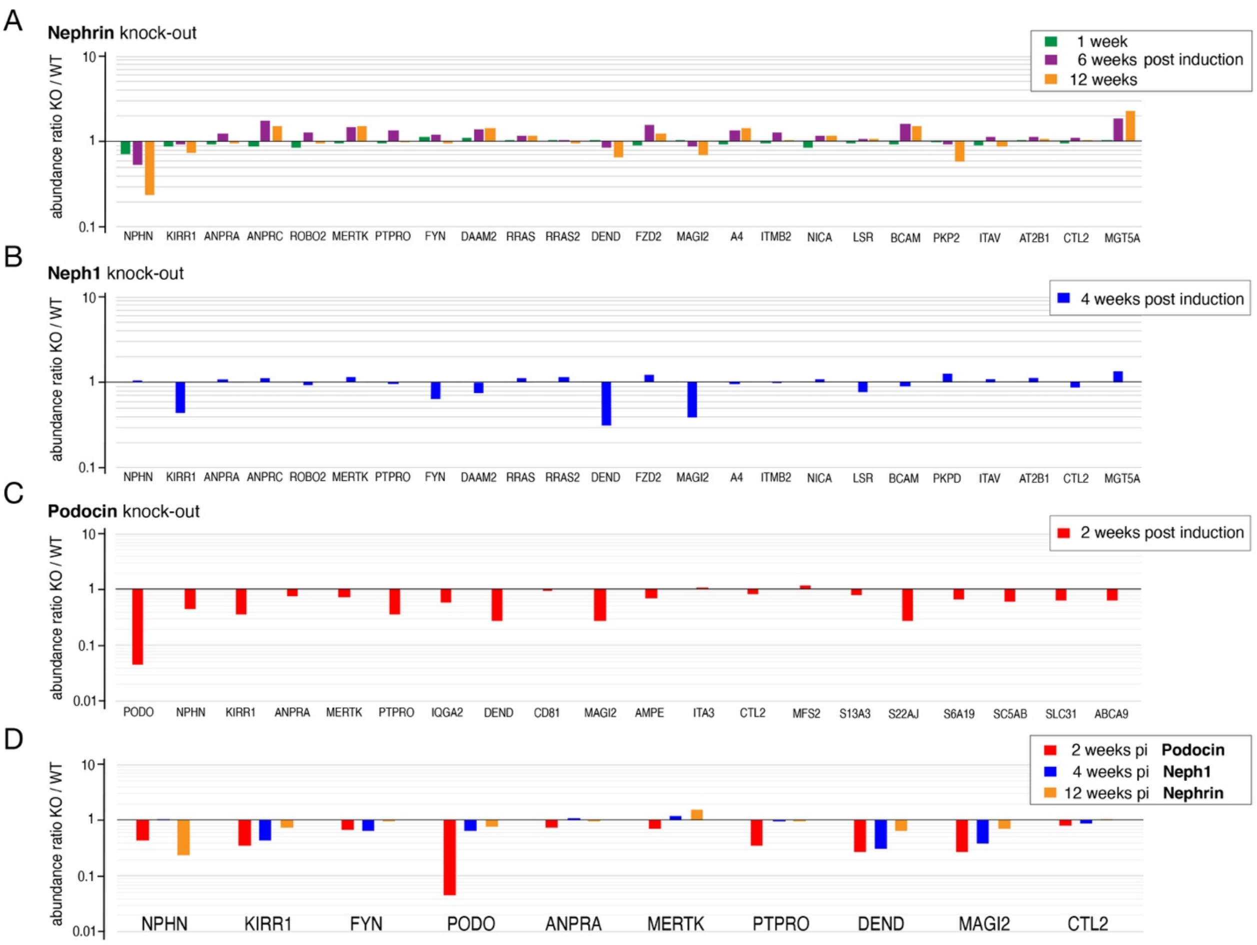

Supplementary Figure 7

Supplementary Figure 8 (next page, information related to Figure 3)

Changes in Nephrin phosphorylation after induction of its knock-down
Results from total membrane analysis (Fig. S7) were inspected for changes in intensity of peptides derived from the C-terminal phosphorylation cluster of Nephrin (Fig. S4, sequence RLAEELSEKTEAGSEEDRLR) 1 and 6 weeks after induction of Nephrin KO ('KO'). Control was without induction ('WT'). (**A**), Abundance ratios as in Fig. S7 for the indicated non-phosphorylated ('non-phospho') and phosphorylated ('phospho') peptides (phosphorylated residues highlighted in red). (**B**), Changes in Nephrin phosphorylation determined by comparing MS-intensity of the indicated phosphorylated peptide species to its non-phosphorylated counterpart in the indicated datasets. Note the roughly 2-fold increase in relative phosphorylation upon induction of Nephrin KO for all three sites/peptides.

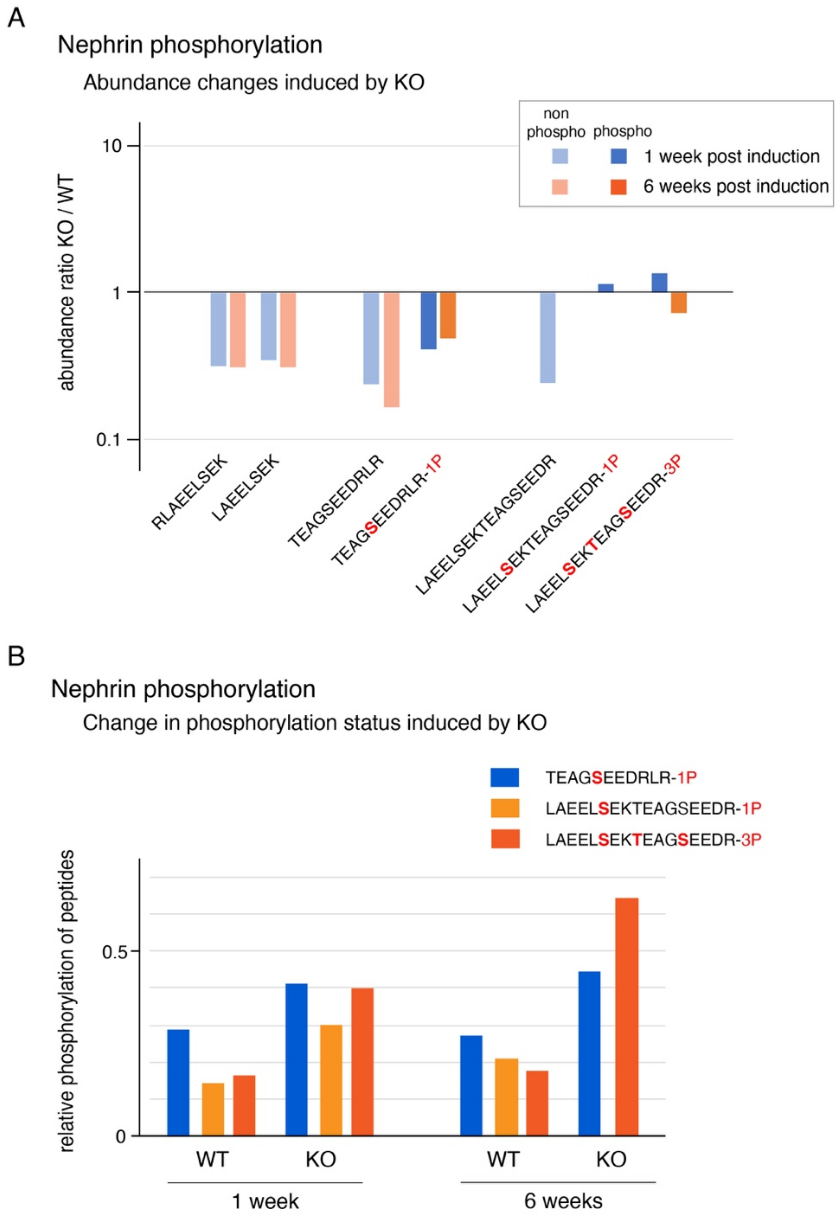

Supplementary Figure 8

Supplementary Figure 9 (next page, information related to Discussion)

Supplementary Figure 9
Enrichment of interactomes with genes causing kidney phenotypes in humans and mice
(**A**), Intersection plot showing overlap between genes in the interactomes determined for Nephrin/Neph1 and Podocin with genes known to cause four different kidney phenotypes in genetically manipulated mice (based on MGI database, http://www.informatics.jax.org/, accessed on July 14, 2021). Genes implicated in abnormal kidney morphology, kidney physiology, and urine protein level were found to be significantly enriched in the Podocin interactome. (**B**), Intersection plot as in (A) but performed with genes known to cause four different subtypes of monogenic kidney disease in humans (based on [1]). Genes implicated

in glomerular kidney disease were found to be significantly enriched in the Podocin interactome. One sided Fisher's exact test was performed in R to test for enrichment of the different gene lists assuming 20,000 total protein-coding genes and a p-value threshold of 0.05/8 = 6.25E-03 to account for multiple testing. Confidence intervals for the odds ratios were computed using the exact2x2 package with tsmethod="minlike" (doi: 10.1093/biostatistics/kxp050). The p-value and odds ratio for each test is in the table, with significant results shown in bold. Odds ratios and p-values are N.A. if no overlap exists. Genes that are common between interactome lists and various kidney phenotype gene lists are labeled with the gene symbol. As negative controls we used genes related to brain size (414 unique genes) and respiratory system development (167 unique genes).

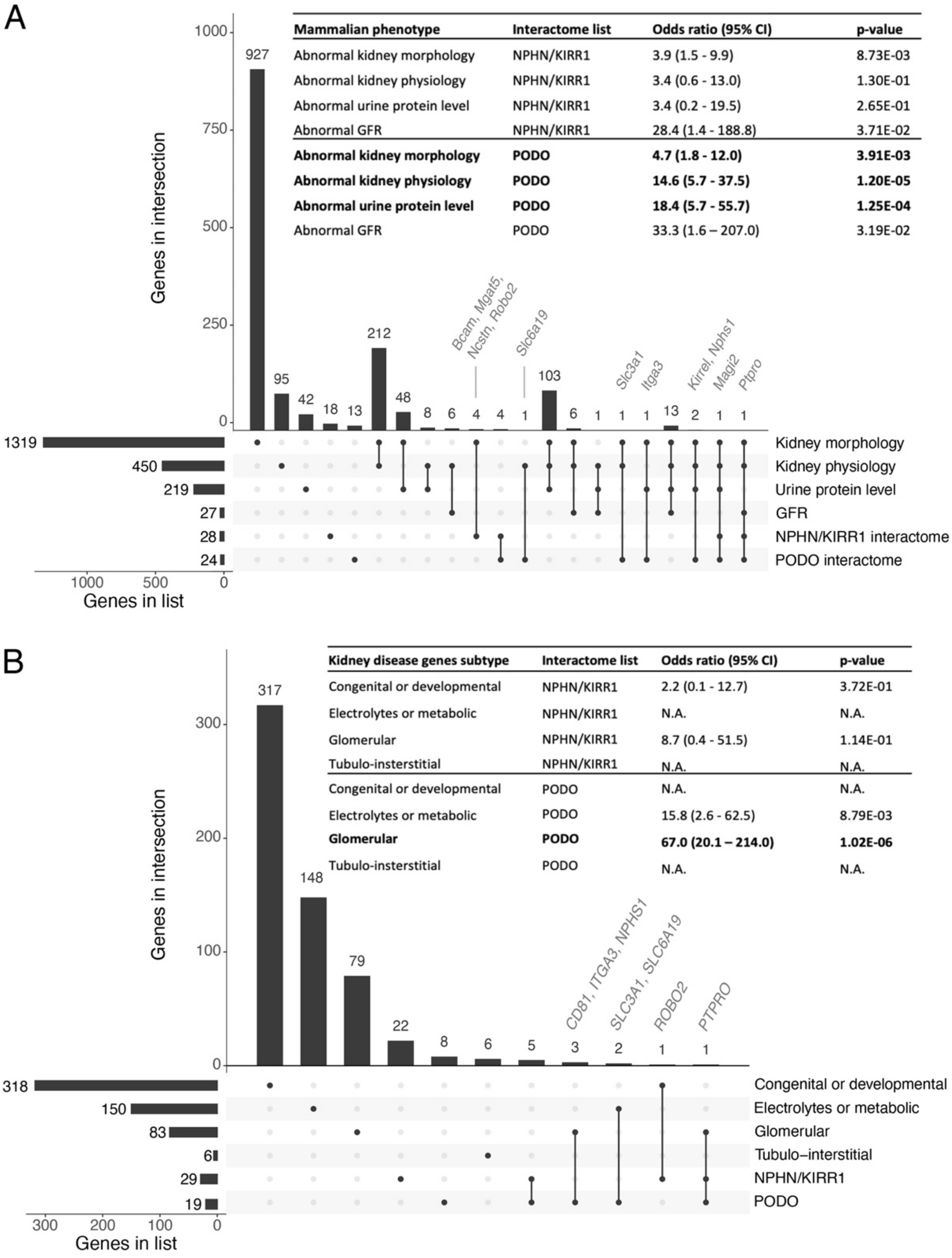

References

1. Rasouly, H.M. *et al.* The Burden of Candidate Pathogenic Variants for Kidney and Genitourinary Disorders Emerging From Exome Sequencing. *Ann Intern Med* **170**, 11-21 (2019).
