# Supplementary material for: A slit-diaphragm-associated protein network for dynamic control of renal filtration
Source: Nat Commun. 2022 Oct 28;13:6446. doi: 10.1038/s41467-022-33748-1 (PMC9616960; doi:10.1038/s41467-022-33748-1)
Supplement: Supplementary file 3 — Description of Additional Supplementary Files [file 41467_2022_33748_MOESM3_ESM.docx]

**Description of Additional Supplementary Files**

**● Supplementary Table
Supplementary Table 1:**  Phosphorylation sites detected in meAP experiments

**● Supplementary Data (related to MS-Analyses)
Supplementary Data 1:** MS-data for determination of the Nephrin/Neph1 interactome
**Supplementary Data 2:** MS-data for determination of the Podocin interactome
**Supplementary Data 3:** MS-data for quantification of membrane fractions from WT and KO mice
