# Supplementary material for: A slit-diaphragm-associated protein network for dynamic control of renal filtration
Source: Nat Commun. 2022 Oct 28;13:6446. doi: 10.1038/s41467-022-33748-1 (PMC9616960; doi:10.1038/s41467-022-33748-1)
Supplement: Supplementary file 8 — Reporting Summary [file 41467_2022_33748_MOESM8_ESM.pdf]

nature portfolio

Corresponding author(s): THuber, FGrahammer, BFakler

Last updated by author(s): September 20 2022

Reporting Summary

Nature Portfolio wishes to improve the reproducibility of the work that we publish. This form provides structure for consistency and transparency in reporting. For further information on Nature Portfolio policies, see our Editorial Policies and the Editorial Policy Checklist.

Statistics

For all statistical analyses, confirm that the following items are present in the figure legend, table legend, main text, or Methods section.

n/a	Confirmed	
☐	☒	The exact sample size (*n*) for each experimental group/condition, given as a discrete number and unit of measurement
☐	☒	A statement on whether measurements were taken from distinct samples or whether the same sample was measured repeatedly
☐	☒	The statistical test(s) used AND whether they are one- or two-sided<br>*Only common tests should be described solely by name; describe more complex techniques in the Methods section.*
☒	☐	A description of all covariates tested
☒	☐	A description of any assumptions or corrections, such as tests of normality and adjustment for multiple comparisons
☐	☒	A full description of the statistical parameters including central tendency (e.g. means) or other basic estimates (e.g. regression coefficient) AND variation (e.g. standard deviation) or associated estimates of uncertainty (e.g. confidence intervals)
☐	☒	For null hypothesis testing, the test statistic (e.g. *F*, *t*, *r*) with confidence intervals, effect sizes, degrees of freedom and *P* value noted<br>*Give P values as exact values whenever suitable.*
☒	☐	For Bayesian analysis, information on the choice of priors and Markov chain Monte Carlo settings
☒	☐	For hierarchical and complex designs, identification of the appropriate level for tests and full reporting of outcomes
☒	☐	Estimates of effect sizes (e.g. Cohen's *d*, Pearson's *r*), indicating how they were calculated

Our web collection on statistics for biologists contains articles on many of the points above.

Software and code

Policy information about availability of computer code

Data collection	ProteoWizard (http://proteowizard.sourceforge.net/) version 3.0.6906, Mascot 2.6., MaxQuant v1.6.3 (http://www.maxquant.org), Xcalibur 2.2 sp1
Data analysis	BELKI software suite (Jordan, J and Haupt, A (2021). Belki: Version 2.0 (v2.0), Zenodo. https://doi.org/10.5281/zenodo.4670785), Microsoft Excel, IGOR Pro 9 (WaveMetrics)

For manuscripts utilizing custom algorithms or software that are central to the research but not yet described in published literature, software must be made available to editors and reviewers. We strongly encourage code deposition in a community repository (e.g. GitHub). See the Nature Portfolio guidelines for submitting code & software for further information.

Data

Policy information about availability of data

All manuscripts must include a data availability statement. This statement should provide the following information, where applicable:

- Accession codes, unique identifiers, or web links for publicly available datasets
- A description of any restrictions on data availability
- For clinical datasets or third party data, please ensure that the statement adheres to our policy

Parts of the MS evaluation work implied the use of the iin-house developed software BELKI. Jordan, J and Haupt, A (2021). Belki9: Version 2.0 (v2.0), Zenodo https://doi.org/10.5281/zenodo.4670785. Database used for evalution MS-data: UniProtKB/Swiss-Prot database (rat, mouse and human entries; release 2019_11, 2022_01)

Human research participants

Policy information about studies involving human research participants and Sex and Gender in Research.

Reporting on sex and gender	N/A
Population characteristics	N/A
Recruitment	N/A
Ethics oversight	N/A

Note that full information on the approval of the study protocol must also be provided in the manuscript.

Field-specific reporting

Please select the one below that is the best fit for your research. If you are not sure, read the appropriate sections before making your selection.

[x] Life sciences [ ] Behavioural & social sciences [ ] Ecological, evolutionary & environmental sciences

For a reference copy of the document with all sections, see nature.com/documents/nr-reporting-summary-flat.pdf

Life sciences study design

All studies must disclose on these points even when the disclosure is negative.

Sample size	The sample size was determined based on previous experience. This relates in particular to the amount of source material for meAP-MS experiments (PMIDs: 22067099, 26691831, 20400944, 31604597), and to the number of mice and flies for analyses of the filtration process (PMIDs: 27297946, 29791858, 11416156, 19713307).
Data exclusions	We did not exclude any data from this study. All relevant data are shown.
Replication	All functional experiments in this study have been replicated using the same experimental setup with similar results; numbers of replications are detailed in the figure legends. Representative results are shown throughout the paper.
Randomization	Flies used for functioinal experiments were randomly selected. Source material for meAP_MS contained all of the pdodocytes/glomerula from multipe WT and KO animals.
Blinding	Evaluation of albumin uptake experiments in drosophila were performed in a blinded fashion as indicated. Mouse experiments could not be performed in a blinded fashion as the condition of highly proteinuric mice had to be closely monitored according to the animal proposal.

Reporting for specific materials, systems and methods

We require information from authors about some types of materials, experimental systems and methods used in many studies. Here, indicate whether each material, system or method listed is relevant to your study. If you are not sure if a list item applies to your research, read the appropriate section before selecting a response.

Materials & experimental systems

n/a	Involved in the study	
	x	Antibodies
x		Eukaryotic cell lines
x		Palaeontology and archaeology
	x	Animals and other organisms
x		Clinical data
x		Dual use research of concern

Methods

n/a	Involved in the study	
x		ChIP-seq
x		Flow cytometry
x		MRI-based neuroimaging

Antibodies

Antibodies used

Complete list of ABs used for meAP-MS is given in Figure S2, additional ABs: anti-Nephrin (Progen #GP-N2, lot 504271, 1:1000), anti-Neph1 (described in 7, 1:250-1:1000), anti-Podocin (Sigma #P0372, lot 035M4851V, 1:1000-2000), anti-ITM2B (Santa-Cruz #sc-50026, 1:200, lot A2407), anti-ATP1A1 (Santa-Cruz #sc-21712, lot 3013, 1:200), anti-HRP-conjugated secondary ABs (Santa-Cruz: sc-2004 (goat anti-rabbit IgG-HRP, lot H01015), 1:25000; sc-2903 (goat anti-guinea pig IgG-HRP, lot I2107, J0812), 1:10000-1:50000; sc-2005 (goat anti-mouse IgG-HRP, lot H02014), 1:25000; sc-2768 (rabbit anti-goat IgG-HRP, lot J0713), 1:50000; Abcam: ab7090 (Goat Anti-Rabbit IgG H&L (HRP) preadsorbed, lot GR270768-25), 1:10000; or Cell Signalling: 7074 (goat Anti-rabbit IgG, HRP-linked Antibody, lot 28), 1:2000), rabbit IgG (Millipore, 12-370, lot 2295402); anti-PODXL (Abcam, ab205350, lot GR268901-3); anti-SLC34A3 (Biorbyt, orb313245, lot BS6202))

Validation

All ABs used for meAPs-based determination of interactomes were validated for the target-specificity and efficiency by quantitative MS (as detailed in Figure S2; ABs with a relative purification-efficiency of > 0.45 were used for maximazing target-to-background ratio (s)). Specificity statements: anti-ATP1A1 (Santa-Cruz #sc-24712: Positive Controls: HeLa whole cell lysate: sc-2200, MDCK cell lysate: sc-2252 or KNRK whole cell lysate: sc-2214) anti-ITM2B (Santa-Cruz #sc-50026: PMID:26691831)

Animals and other research organisms

Policy information about studies involving animals; ARRIVE guidelines recommended for reporting animal research, and Sex and Gender in Research

Laboratory animals

Mice
Mice were housed in a SPF facility with free access to chow and water and a 12 h day/night cycle. Humidity was set by a room specific air conditioning at 45-65% and temperature between 20-24°C. Breeding and genotyping were done according to standard procedures. Nphs1 (kind gift from Alessia Fornoni, Miller University, Miami, USA 54), Nphs2 (kind gift from Géraldine Mollet and Corinne Antignac, Imagine Institute of Genetic Diseases, Paris, France 55) and Neph1 7 floxed mice all on a C57Bl6/Crl background, were crossed with Tg(Nphs1-rtTA*3G)8Jhm (kind gift from Jeff Miner, Washington University, St. Louis, USA) and Tg(tetO-cre)1Jaw (Jackson Laboratory, USA) yielding Nphs1ΔiPod, Nphs2ΔiPod and Neph1ΔiPod respectively. All mice were used for experiments at an age of 5 weeks. Male and female mice were used with equal proportions thereby complying with German animal legislation. Doxycyclin (2mg/ml in 5% sugar solution, Fagron, Barsbüttel, Germany) was administered for 8 days in the drinking water at an age of 5 weeks.

Rats
CD1 male rats were bought at 7 weeks from Charles River, Germany and after arrival were housed for one week in a SPF facility with free access to chow and water and a 12 h day/night cycle. Humidity was set by a room specific air conditioning at 45-65% and temperature between 20-24°C. Afterwards rats were killed for glomerular isolation.

Drosophila
D. melanogaster stocks were cultured on standard cornmeal molasses agar food and maintained at 25°C. The Drosophila homologues of mammalian genes were identified using the DIOPT DRSC Integrative Ortholog Prediction Tool (Harvard Medical School) 59. RNAi-Based Nephrocyte Functional Screen Procedure: Virgins of prospero-Gal4 (gift from Barry Denholm, University of Edinburgh, Edinburgh, UK), MHC-ANF-RFP, HandGFP and Dot-Gal4 transgenic lines (gift from Zhe Han, University of Michigan, Ann Arbor, USA) were crossed to UAS-CG3653-RNAi (KIRRE, NEPH1, VDRC 27227/GD); UAS-CG3662–RNAi (ITM2B, Bloomington Drosophila Stock Center 18281); UAS-CG2272-RNAi (MERTK, VDRC 33518/GD); UAS-CG14877-RNAi (NPR3, VDRC 45324/GD); UAS-CG1725-RNAi (DLG1, VDRC 109274/KK) males at 25°C. Two days after crossing, flies were transferred to small collection cages with grape juice agar plates to collect the embryos after 24 hours at 25°C. Collected embryos were aged for 48 hours at 29° C. Nephrocytes of L3 larva were dissected. At this timepoint no differentiation between male and female larvae could be visually performed, hence both sexes were used.

Wild animals

No wild animals were used in this study.

Reporting on sex

As fundamental biological processes were studied sex differences were not deemed to be of any relevance for the primary notion of this manuscript. As indicated mice of both sexes with equal proportions were used, yet the study was not powered enough to report results in a sex specific manner. Due to availability only male rats were used in this study, again sex was not deemed relevant for the renal filtration process per se. As described for drosophila larvae from both sexes were used as sex could not be determined at this developmental stage.

Field-collected samples	No field collected samples were used in this study.
Ethics oversight	This study was performed according to the animal proposal G14/22 issued by the Regierungspräsidium Freiburg, Freiburg, Germany.

Note that full information on the approval of the study protocol must also be provided in the manuscript.
